# Comparison of Heavy Labeled (SIL) Peptide versus SILAC Protein Internal Standards for LC-MS/MS Quantification of Hepatic Drug Transporters

**DOI:** 10.1155/2014/451510

**Published:** 2014-02-25

**Authors:** Bhagwat Prasad, Jashvant D. Unadkat

**Affiliations:** Department of Pharmaceutics, University of Washington, P.O. Box 357610, Seattle, WA 98195, USA

## Abstract

We studied the precision of quantification of organic anion-transporting polypeptide 1B1 (OATP1B1), OATP1B3, OATP2B1, and P-glycoprotein (P-gp) in human livers by surrogate peptide based LC-MS/MS approach using two different internal standards: stable isotope labeled peptide (SIL) versus stable isotope labeled protein (SILAC). The SIL peptides were procured commercially and the SILAC proteins were generated in-house by labeling arginine and/or lysine residues in cells expressing these transporters. Liver tissue (n = 20) was homogenized and the membrane fraction was isolated. The membranes were trypsin digested and the peptides were analyzed using LC-MS/MS under optimized conditions. The precision in the quantification of proteins in three independently trypsin digested samples from each liver was calculated as the standard deviation of the log transformed protein concentration. The precision of the SIL internal standard method was either slightly (*P* < 0.05, paired *t*-test) better than that of the SILAC method (OATP1B1, OATP1B3, and P-gp) or not different (OATP2B1). Trypsin digestion, as measured by the response of the labeled peptide derived from the SILAC protein, was consistent across liver samples. These results indicate that when maximum trypsin digestion is ensured, the SIL internal standard method can be used with confidence for quantification of drug transporters.

## 1. Introduction

Signature peptide based quantification of proteins using liquid chromatography-tandem mass spectrometry (LC-MS/MS) is increasingly used to measure endogenous or therapeutic proteins because it is a high-throughput, selective, and sensitive method when compared with other quantitative proteomics and immunoblotting methods [1]. In this approach, the analyte protein is first digested, the peptides are separated by LC, and the unique peptide representing target protein is analyzed by MS in multiple reaction monitoring (MRM) mode [2, 3]. The protein abundance is typically measured based on calibration curve generated using the unique synthetic peptide as an external standard and the stable isotope labeled peptide (SIL) as an internal standard [3]. Although this approach has been validated for LC-MS/MS variability [2, 3], variation in trypsin digestion is not addressed by this approach [4]. Conventionally, trypsin digestion conditions (e.g., incubation time and trypsin : protein ratio) that ensure maximum protein digestion are established to minimize the influence of such variability [5–7]. However, even under these circumstances, the sample or process dependent factors [8] can cause variability in protein digestion. As a result, relying on the SIL peptide as an internal standard can be questioned. In such cases, in theory, the use of the whole stable isotope labeled protein (SILAC) as an internal standard would be considered to be superior to the use of SIL peptide [4, 9–13]. Therefore, we investigated if the use of SILAC protein as an internal standard would result in greater assay precision than using the SIL peptide. We compared these methods using four hepatic drug transporter proteins, that is, organic anion transporter polypeptide (OATP) 1B1, OATP1B3, OATP2B1, and P-glycoprotein (P-gp).

It is important to note here that our method differs in a fundamental way from the “absolute SILAC” method proposed by Hanke et al. [12]. In that method, the labeled SILAC protein is used as a calibrator while it is used as an internal standard in our method. Because Hanke et al. used the SILAC protein as a calibrator, the quantity of the protein used must be known and therefore it must be purified before use. While this approach is ideal and takes into consideration variability in trypsin digestion, it cannot be used to quantify expression of membrane proteins (e.g., transporters) because purified recombinant versions of these proteins are extremely difficult to routinely obtain. Therefore, our approach utilizes synthetic pure peptides as calibrators and the labeled SILAC protein as the internal standard. Since the SILAC protein is used only as an internal standard, it does not need to be pure though it must be completely or almost completely labeled (i.e., isotopically pure).

## 2. Materials and Methods

### 2.1. Chemicals and Reagents

Heavy amino acids, ^13^C_6_^15^N_2_-lysine and ^13^C_6_^15^N_4_-arginine, were obtained from Sigma-Aldrich (St. Louis, MO). Low-glucose Dulbecco's modified Eagle's medium (DMEM), phosphate-buffered saline (PBS), penicillin/streptomycin, and geneticin were purchased from Invitrogen, Carlsbad, CA. Fetal bovine serum (FBS) and dialyzed FBS were procured from Gemini Bio-Products, Calabasas, CA. SILAC DMEM (without L-lysine and L-arginine) was purchased from Thermo Fisher Scientific, MA, USA. The ProteoExtract Native Membrane Protein Extraction Kit was purchased from Calbiochem (Temecula, CA). The protein quantification BCA kit and the in-solution digestion kit were from Pierce Biotechnology (Rockford, IL). Pure synthetic unlabeled peptides, NVTGFFQSFK, NVTGFFQSLK, VLAVTDSPAR, and NTTGALTTR, with purity determined by quantitative amino acid analysis, were obtained from New England Peptides (Boston, MA). The SIL internal standards, NVTGFFQSF[^13^C_6_^15^N_2_]K, NVTGFFQSL[^13^C_6_^15^N_2_]K, VLAVTDSPA[^13^C_6_^15^N_4_]R, and NTTGALTT[^13^C_6_^15^N_4_]R, were from Thermo Fisher Scientific (Rockford, IL). HPLC-grade acetonitrile and other solvents were purchased from Fischer Scientific (Fair Lawn, NJ) and formic acid was purchased from Sigma-Aldrich (St. Louis, MO).

### 2.2. Human Liver Samples and Cell Lines

Twenty human liver samples (HL1-HL20) were obtained from Human Liver Bank of the University of Washington (UW) School of Pharmacy. All the subjects were Caucasians; age 25–67 yr; 11 female and 9 male. Procurement, characteristics, and storage of these liver samples has been previously described [14, 15]. Due to the anonymous nature of these samples, their use was classified as non-human subjects research by the UW Human Subjects Division. P-gp-expressing LLCPK-MDR1 cells were kindly provided by Dr. Alfred Schinkel (The Netherlands Cancer Institute, Amsterdam, The Netherlands). Human OATP1B1, OATP1B3, and OATP2B1 expressing HEK293 cells were kindly provided by Dr. Markus Keiser (Department of Clinical Pharmacology, University of Greifswald, Germany).

### 2.3. Cell Culture and SILAC Labeling

The P-gp-expressing LLCPK-MDR1 cells were grown in DMEM supplemented with 10% FBS, 100 U/mL penicillin, and 100 *μ*g/mL streptomycin at 37°C under 5% CO_2_ and 95% humidity. The individual OATP1B1, OATP1B3, and OATP2B1 expressing HEK cells were grown using PDL-coated flasks for better cell adhesion and growth. Geneticin (G-418 Sulfate, Invitrogen) (750 *μ*g/mL) was used as selection antibiotic in the media. SILAC labeling of the proteins was as per a previous protocol [9]. Briefly, stock solutions of ^13^C_6_^15^N_2_-L-lysine and ^13^C_6_^15^N_4_-L-arginine were prepared in PBS at concentrations of 146 mg/mL and 84 mg/mL, respectively. 0.5 mL of the stock solutions was then added to 1 liter SILAC DMEM containing FBS and antibiotics as discussed above. The SILAC labeling was performed using exactly the same conditions as used for normal cell culture except the normal medium was replaced with the SILAC medium. After approximately five doublings of the cells, ~10^5^ cells were isolated and subjected to membrane extraction. Trypsin digestion was performed of the membrane fraction to quantify the extent of labeling by comparing MS response for three labeled and unlabeled peptides in full scan MS mode for each protein [9]. The cells were exposed to the SILAC medium until more than >95% of the protein was labeled. Later, the cells were harvested and cell pellet was stored at −80°C before membrane extraction.

### 2.4. Membrane Protein Extraction and Total Protein Quantification

The liver tissue (~100 mg) was processed to isolate the membrane fraction as per manufacturer's instructions (Calbiochem, Temecula, CA) and the previously described method [15–17]. Briefly, the tissue was subjected to homogenization in 2 mL extraction buffer I (EB-I; ProteoExtract Native Membrane Protein Extraction Kit) plus protease inhibitor cocktail of the kit and incubated with gentle shaking for 10 min. The homogenate was centrifuged at 16,000 ×g for 15 min and the supernatant was removed. The pellet was resuspended in 1 mL EB-II (containing surfactants) from the kit plus 10 *μ*L of protease inhibitor cocktail. The suspension was incubated with gentle shaking for 30 min at 4°C followed by centrifugation at 16,000 ×g for 15 min at 4°C. Total membrane protein concentration in the isolated membrane fraction (i.e., supernatant) was determined using the BCA protein assay kit and diluted to a working concentration of 2 *μ*g/*μ*L. Similar to the tissues, the labeled cell pellet (2–5 × 10^6^ cells) was processed as discussed above except that the cells were washed twice with PBS before adding 2 mL of the EB-I to the cells followed by gently shaking for 10 min. The remaining procedure was as described for the tissue.

### 2.5. In Silico Peptide Selection, Trypsin Digestion of Membrane Protein, and Sample Preparation

The signature peptides were selected based on online in silico prediction tools [2] as well as the literature [4, 15] (Table 1). Briefly, peptides susceptible to degradation, that is, containing methionine, cysteine, histidine, and tryptophan, were not selected. Peptides within the transmembrane regions or containing single nucleotide polymorphism and posttranslational modifications were not considered. Continuous sequences of R and K (RR, RK, KR, KK) were avoided in the region of trypsin digestion to avoid miscleavages. The length of selected peptides was from 8 to16 amino acid residues. A genome wide BLAST search was also performed to ensure that the peptide was selective for the protein. Only one peptide per protein was selected in this study because our goal was to compare the precision of SIL peptide versus SILAC protein internal standards. The indicated peptide (Table 1) was selected over other possible signature peptides, because it yielded the best signal to noise ratio in the liver membrane samples.

**Figure 1:**
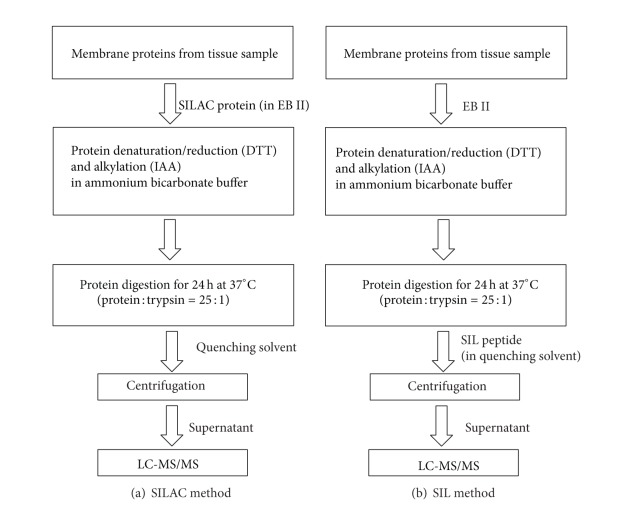
Comparison of SILAC versus SIL internal standard methods. SILAC protein is added before trypsin digestion, while SIL peptide is added just before LC-MS analysis. Key: DTT, dithiothreitol; IAA, iodoacetamide; EB-II, extraction buffer II.

Slightly different sample preparation conditions were used for SIL versus SILAC internal standard methods prior to LC-MS analysis as shown in Figure 1. For trypsin digestion, the optimized protein : trypsin ratio was maintained at 25 : 1 for both methods. Before trypsin digestion, the labeled cell membrane extracts were assessed for protein expression and pooled to prepare a cocktail of labeled OATP1B1, OATP1B3, OATP2B1, and P-gp. For the SILAC method, 20 *μ*L of 2.0 *μ*g/*μ*L of tissue membrane fraction and 12 *μ*L of 0.67 *μ*g/*μ*L of labeled cell membrane fraction were denatured with 4 *μ*L dithiothreitol (100 mM) and alkylated with 4 *μ*L iodoacetamide (200 mM) in 10 *μ*L ammonium bicarbonate digestion buffer (50 mM, pH 7.8) using the previously outlined protocol [15, 17]. The protein samples were digested by trypsin (10 *μ*L) in a final volume of 60 *μ*L at 37°C for 24 h and the reaction was quenched by 30 *μ*L of quenching solvent (70% acetonitrile in water containing 0.1% formic acid). Samples were centrifuged at 4000 ×g for 5 min and supernatant was used for LC-MS/MS analysis.

For the SIL internal standard method, the synthetic SIL peptide was used as internal standard. 20 *μ*L of 2.0 *μ*g/*μ*L total membrane protein was trypsin digested as described above except that EB II was used instead of the SILAC protein. The reaction was quenched by 20 *μ*L of SIL peptide internal standard cocktail (prepared in the quenching solvent described above) and 10 *μ*L of the neat quenching solvent. The samples were centrifuged as described above. For both methods (SIL and SILAC), signature peptide standards were used calibrators. The calibration curves were generated by using 20 *μ*L of EB II instead of 20 *μ*L of the tissue membrane protein in samples and the neat quenching solvent above was replaced with the signature peptide cocktail.

### 2.6. UHPLC-MS/MS Parameters

The UHPLC-MS/MS system consisting of Agilent 6460A triple-quadrupole mass spectrometer coupled to Agilent 1290 Infinity LC system (Agilent Technologies, Santa Clara, CA) was operated in ESI positive ionization mode. For quantification, the dynamic MRM algorithm was used to maximize dwell time on each transition to allow multiplexed quantification. Approximately 2–2.5 *μ*g of the digest (5 *μ*L) was injected onto the column (Kinetex 2.6 *μ*m, C18, 100 × 3 mm, Phenomenex, Torrance, CA) and eluted at 0.4 mL/min by a mobile phase with initial conditions of 97% A (water containing 0.1% v/v formic acid) and 3% B (acetonitrile containing 0.1% v/v formic acid) held for 4 min, followed by seven steps of linear gradient of mobile phase B concentration of 3% to 12.5%, 12.5% to 18%, 18% to 19.5%, 19.5% to 20%, 20% to 35%, 35% to 50%, and 50% to 90% over 4–8 min, 8–11 min, 11–13.5 min, 13.5–16 min, 16–18 min, 18–18.4 min, and 18.4–18.6 min. Then, the column was washed using 90% mobile phase B for 1.6 min followed by a reequilibration period of 4.8 min. The doubly charged parent to singly charged product transitions for the analyte peptides and their respective labeled peptides were monitored. The LC-MS/MS parameters are shown in Table 1.

### 2.7. Method Validation and Sample Analysis

SIL internal standard method was validated for lower limit of quantification, linearity, range, accuracy, precision, and stability. The calibration curve was generated using 7 calibrators, ranging 0.1–6.0 fmol/*μ*g of total digested protein for OATP1B1, OATP1B3, OATP2B1, and P-gp, respectively. The calibration curve standards were prepared by spiking peptide standards into the EB II. The LC-MS injection volume was 5 *μ*L. Assay accuracy and precision was performed in triplicate at three different quality control (QC) concentrations (low, middle, and high) of each peptide across the calibration range. Two different matrices were used, that is, pooled human liver membrane protein (*n* = 50) or EB II. Although MRM data were acquired using three different transitions, only the two most intense transitions (Table 1) were processed by integrating the peak areas generated from the reconstructed ion chromatograms for the analyte peptides and their respective internal standards using the MassHunter software (Agilent Technologies, Santa Clara, CA). The average peak areas of these two MRM transitions were used for the calibrators and internal standard (SIL or SILAC) to construct the calibration line and to estimate the transporter protein concentration in the unknown samples.

The impact of freeze-thaw stress on protein quantification was assessed by exposing liver tissue membrane extracts (*n* = 3) to zero or three freeze and thaw cycles before trypsin digestion. Similarly, the effect of bench-top stability on protein quantification was investigated by storing the membrane preparation at ambient temperature for 6 h prior to trypsin digestion. Additionally, the autosampler stability of the peptide was determined by repeating analysis of the extracted samples, stored in the LC-MS autosampler (at 6°C), over 48 hr. The SILAC internal standard method was also validated for all the parameters described for SIL internal standard method except stability, which was common for both methods.

Additionally, to ensure maximum trypsin digestion, membrane fraction isolated from a pooled human liver sample was subjected to digestion in triplicates up to 24 h (1, 2, 5, 16, and 24 h) as described above. After 24 h, fresh trypsin was added to the samples and incubated for another 24 h. The magnitude of protein digestion in all samples was expressed relative to that in the 24 h samples.

Finally, protein expression in 20 liver samples was determined in triplicate using the two internal standard methods.

### 2.8. Data Analysis

Since 100% SILAC labeling is rarely achieved, the concentration of SILAC protein as internal standard was kept low to minimize the effect of endogenous unlabeled protein on quantification. In addition, the endogenous unlabeled protein response of SILAC protein was taken into consideration by subtracting it from the analyte response. Similarly, the endogenous protein expression in QC samples, which were prepared by spiking standards into the pooled liver membrane matrix, was also taken into consideration. The precision of the two methods (SILAC versus SIL) was compared by the paired *t*-test analysis of the standard deviation of protein expression in the three independently trypsin digested samples from each liver. Prior to statistical analysis, the data were log transformed as they were found to be log-normally distributed. The individual and population mean ± SD protein expression across the 20 livers was also computed.

## 3. Results and Discussion

### 3.1. UHPLC-MS/MS Method Development and Validation

**Figure 2:**
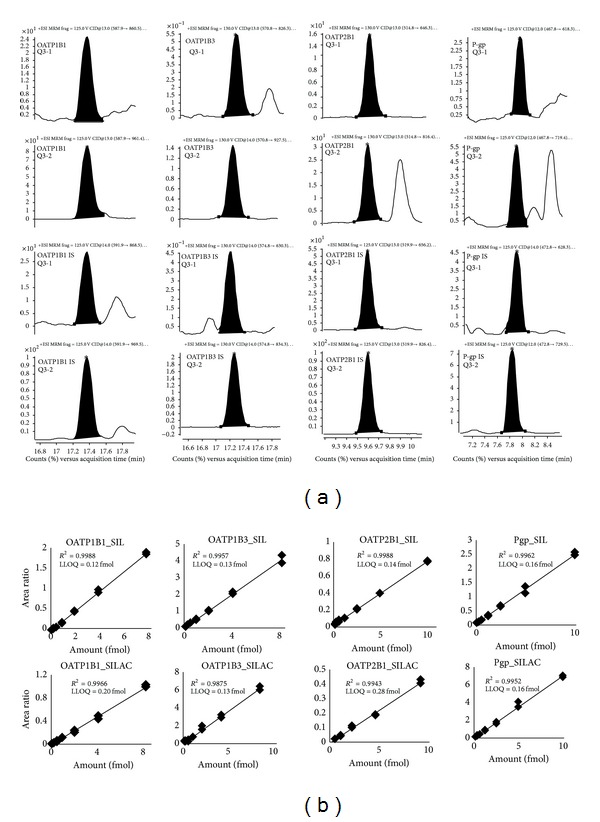
LC-MRM chromatograms of a representative trypsin digest of liver tissue membrane sample. The chromatograms show elution patterns and selectivity for P-gp, OATP2B1, OATP1B3, and OATP1B1 peptides (Table 1), respectively (left to right). Q3-1 and Q3-2 represent two different MRM transitions of a peptide. The top two channels show analyte peptides and the bottom two channels show internal standards (IS) (a). Representative calibration curves (peak area calibrator/internal standard versus amount on-column of the calibrator) and LLOQs (femtomoles, on-column) of OATP1B1, OATP1B3, OATP2B1, or P-gp (b). Limits of detection (LODs) were 3–5-fold lower than LLOQs.

**Figure 3:**
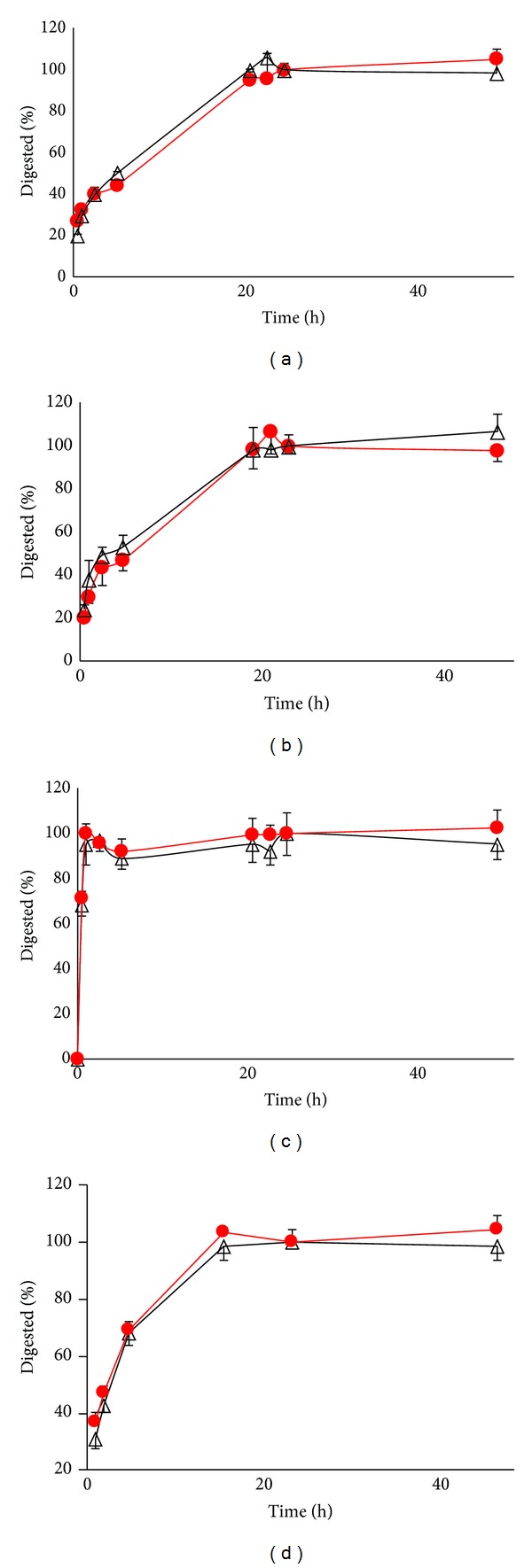
Comparison of formation of the monitored peptides (unlabeled (∆) and labeled (filled circle)) during trypsin digestion of OATP1B1 (a), OATP1B3 (b), OATP2B1 (c), and P-gp (d). Data are presented as mean ± SD. At 24 h, additional trypsin was added (see method). Data are as a percent of trypsin digestion at 24 h (mean ± of *n* = 3).

LC-MS/MS chromatograms (Figure 2) show the specificity of the analytical method. The calibration curves generated using both SIL and SILAC internal standard methods showed linear response throughout the range. The lower limit of quantification, defined as the lowest concentration of spiked peptides in pooled human liver membrane fraction with error and precision less than or equal to 25%, was 0.13, 0.08, 0.05, and 0.10 fmol/*μ*g digested protein for OATP1B1, OATP1B3, OATP2B1, and P-gp, respectively. Accuracy and precision (% coefficient of variance (%CV)) in the quantification of the QC samples were found to be acceptable (Table 2) at the three different concentrations using both the MRM transitions (Table 1) as per FDA bioanalytical method validation guideline for proteins immunoquantification [18]. Rate of protein digestion of unlabeled versus SILAC OATP1B1, OATP1B3, OATP2B1 and P-gp was parallel (Figure 3). Optimum trypsin digestion was confirmed by comparing peptide recovery at 24 h versus 48 h. The peptide recovery after 48 h did not change significantly (Figure 3). It is important to note here that maximum digestion may not be equivalent to complete digestion. Test of complete digestion is possible only when pure protein standards for these transporters are available. Although three of the four peptides selected contain a potentially labile asparagine residue at the N-terminus, the peptide response was stable even when sample was exposed to three freeze-thaw cycles and at bench-top for 6 h (Table 3). This indicates that sample processing variables do not affect quantification of these transporters using selected peptide approach. The peptides were also inherently stable for 48 h in the autosampler used.

### 3.2. Transporter Quantification Using SILAC Protein or SIL Peptide Internal Standards

**Figure 4:**
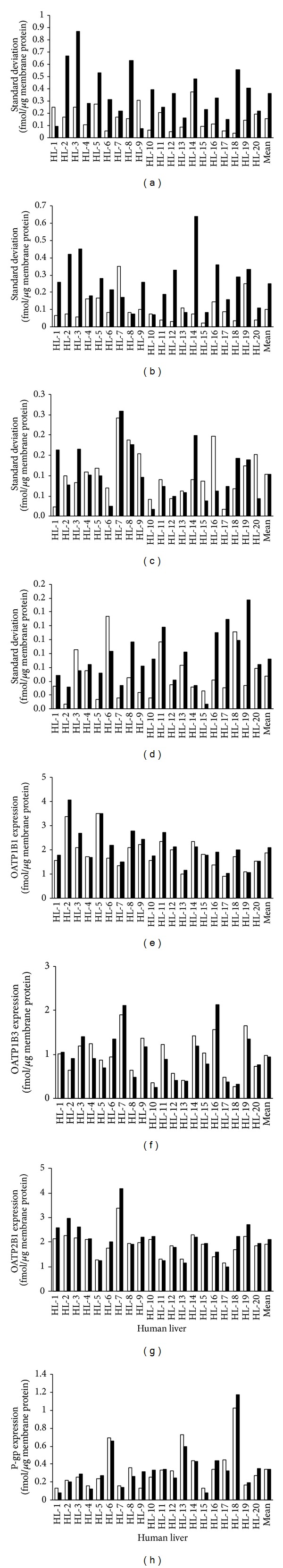
Standard deviation (a to d) and expression (e to h) of OATP1B1, OATP1B3, OATP2B1, and P-gp in individual liver sample (mean of triplicates) as determined by SIL (open bars) or SILAC (filled bars) internal standard methods. The last bar represents the population mean value. The individual hepatic expression data using SIL method are from our previous publication [15].

The mean and range of standard deviation of triplicate measurements of OATP1B1, OATP1B3, and P-gp in 20 liver tissue samples were marginally (but significantly) larger with the SILAC versus the SIL internal standard method (Figure 4, Table 4). In other words, the SIL method was slightly more precise than the SILAC method when quantifying the hepatic expression of OATP1B1, OATP1B3, and P-gp. For quantification of OATP2B1, the precision of the two methods was not significantly different (Figure 4, Table 4). As assessed by the paired Student's *t*-test, the population mean (±SD) protein expression (fmol/*μ*g of membrane protein) in twenty livers estimated by the SIL versus SILAC method was not significantly different for OATP1B3 (0.97 ± 0.47 versus 0.94 ± 0.54) or P-gp (0.34 ± 0.23 versus 0.34 ± 0.25) but was marginally different for OATP1B1 (1.86 ± 0.68 versus 2.07 ± 0.77) and OATP2B1 (1.91 ± 0.50 versus 2.10 ± 0.73).

Hence, in contrast to the theoretical prediction, we found that the SIL internal standard method was either marginally more precise than the SILAC method or the precision of the two methods was not significantly different (Figure 4 and Table 4). Consistent with this observation, the variability in the QC samples was also higher when using the SILAC versus SIL internal standard approach (Table 2). This was perhaps due to the small MS response of these low abundant transporters, where variability, introduced due to the additional biological matrix present in the SILAC internal standard method, exceeded the variability in trypsin digestion. The latter was consistent across samples as measured by the standard deviation of the LC-MS/MS response of the labeled peptide originating from SILAC protein. The above statement is supported by our observation that the precision in determination of expression of the highly MS responsive protein, OATP2B1 (Table 4), was not different between the two methods. Surprisingly, the estimate of the mean value of transporter expression in each liver sample was also found to be marginally but significantly different (see above). The SILAC internal standard method reported marginally higher expression for two of the four transporters (OATP1B1, OATP2B1), perhaps due to the unlabeled protein in the SILAC internal standard. Based on these data, when trypsin digestion is maximized and all other constituents are the same (e.g., the matrix) and the conditions are strictly controlled (e.g., protein concentration), we predict that SIL internal standard method will perform as good or better than the SILAC internal standard method.

The population mean transporter expression in human liver in our study was comparable or modestly lower than those reported by other groups [19–22]. However, unless confirmed by pure protein standards, the limitation of these peptide based LC-MS/MS quantification methods is the assumption of complete trypsin digestion. To address the latter, we recently validated the surrogate peptides based protein quantification method using the only available purified transporter available to us, namely, P-gp. When this purified transporter was used as QC sample, the recovery of quantity in these P-gp quality control samples was within 73–125% of the actual value [15]. The mean expression and variability of individual transporters by the two methods are presented in Figures 4(e) to 4(h). The individual hepatic expression data are from our previous publication [15].

## 4. Conclusions

Metabolic labeling of proteins by labeled amino acids, that is, SILAC, is an established technique for relative quantification of proteins [23–26] and has also been used for quantification of proteins [4, 9–11]. As outlined in the introduction, use of labeled protein generated using SILAC approach is considered better in peptide based LC-MS/MS protein quantification because the SIL method cannot take into account any variability in trypsin digestion. Since trypsin digestion is expected to be affected by different sample and process dependent variables [8], the SIL method is theoretically expected to be less precise than the SILAC method. However, the accuracy of the two methods is expected to be equivalent as the two methods differ only in the internal standard used. Here, we tested for the first time these theoretical predictions. Our study concludes that if trypsin digestion is consistent across samples, the precision of the SIL method in quantification of proteins is similar to SILAC internal standard method. Moreover, generating the labeled protein is a time and cost intensive. Thus, we recommend that the SIL method be used for quantification of proteins when a single matrix is used. Whether this conclusion will remain the same when different biological matrices (e.g., liver versus kidney or different sample extraction buffers) are used remains to be tested. Quantification of transporter expression in various human tissues, including the interindividual variability in expression due to age, sex, or genotype, will be invaluable for prediction of in vivo disposition of drugs from in vitro data. Such studies are ongoing in our laboratory.

## Figures and Tables

**Table 1:** MRM parameters of peptide selected for targeted analysis of OATP1B1, OATP1B3, OATP2B1, and P-gp. The labeled amino acid in the internal standard is shown in bold and italic.

Transporter	Peptide	Parent ion	Product ions		Fragmentor (V)	Collision energy (eV)
			1	2		
OATP1B1	NVTGFFQSFK	587.9	961.4	860.5	125	13
	NVTGFFQSF***K***	591.9	969.5	868.5	125	14

OATP1B3	NVTGFFQSLK	570.8	927.5	826.3	130	14
	NVTGFFQSL***K***	574.8	935.6	834.3	130	14

OATP2B1	VLAVTDSPAR	514.8	816.4	846.4	130	13
	VLAVTDSPA***R***	519.9	826.4	856.4	125	13

P-gp	NTTGALTTR	467.8	618.3	719.4	125	12
	NTTGALTT***R***	472.8	628.3	729.5	125	12

Isotopic purity of synthetic SIL peptides was 100%. SILAC protein labeling was achieved up to 95.8% (OATP1B1), 98.1% (OATP1B3), 97.2% (OATP2B1), and 98.2% (P-gp).

**Table 2:** Accuracy and precision (%CV) of LC-MS/MS method of quantification of OATP1B1, OATP1B3, OATP2B1, and P-gp using SIL or SILAC internal standard method.

Transporters	Matrix	QC level	Amount (fmol, on-column)	SIL internal standard (*n* = 3)				SILAC internal standard (*n* = 3)			
				Transition 1		Transition 2		Transition 1		Transition 2	
				Accuracy	%CV	Accuracy	%CV	Accuracy	%CV	Accuracy	%CV
OATP1B1	EB-II^#^	Low	0.9	112.6	4.6	87.6	13.6	84.4	16.9	93.4	12.7
		Middle	6.3	93.4	13.2	107.8	4.7	98.5	12.5	109.5	13.5
		High	12.5	12.2	5.9	95.8	12.4	111.4	6.7	116.9	14.9
	HLP*	Low	2.3	110.3	12.3	88.9	11.2	119.2	12.9	97.3	19.7
		Middle	6.3	106.2	6.4	109.8	14.6	92.3	15.2	104.8	12.5
		High	12.5	105.5	4.5	97.8	5.6	116.2	9.8	106.5	16.8

OATP1B3	EB-II	Low	1.0	86.3	16.8	117.8	14.3	78.8	19.9	93.5	11.6
		Middle	6.7	106.4	6.7	93.7	13.3	87.3	13.6	102.4	7.9
		High	13.3	111.8	6.8	84.4	12.2	102.4	8.9	119.4	11.9
	HLP	Low	2.3	102.6	6.6	104.6	15.4	122.1	18.4	84.3	23.0
		Middle	6.7	103.3	7.5	111.9	10.4	126.3	14.6	99.4	17.8
		High	13.3	94.5	14.9	118.6	6.8	112.4	7.9	112.8	8.8

OATP2B1	EB-II	Low	0.7	84.3	9.4	83.5	5.6	82.8	14.4	122.9	12.5
		Middle	4.6	105.5	2.8	91.9	13.4	114.0	17.8	95.6	12.5
		High	9.2	92.6	12.4	104.8	8.1	103.5	9.8	104.5	15.7
	HLP	Low	1.6	109.7	14.6	109.7	15.1	108.4	12.6	88.3	14.6
		Middle	4.6	114.5	8.5	103.4	8.2	119.5	11.3	86.5	9.8
		High	9.2	95.8	8.7	91.6	2.5	122.3	14.7	109.7	12.9

P-gp	EB-II	Low	1.2	106.2	5.8	94.2	12.4	120.1	13.9	89.4	19.5
		Middle	8.1	93.3	8.3	113.1	8.9	123.5	12.4	105.4	12.7
		High	16.1	109.9	10.1	112.4	6.4	107.9	13.6	91.3	13.9
	HLP	Low	2.8	117.7	11.1	91.3	16.2	118.4	25.5	123.6	18.8
		Middle	8.1	94.9	9.4	116.7	13.5	97.2	17.4	106.9	9.9
		High	16.1	101.9	11.7	109.3	12.3	117.2	6.4	103.4	7.8

Mean				98.8	9.1	101.7	10.7	107.8	13.6	102.8	13.8
SD				20.0	3.6	10.9	3.8	14.0	4.3	11.1	3.8

^#^EB-II: extraction buffer II; *HLP: human liver membrane pool. For method validation, 20 *μ*L of EB-II or HLP (2 mg/mL) was diluted to 90 µL (see Section 2 for details).

**Table 3:** Bench-top and freeze-thaw stability of OATP1B1, OATP1B3, OATP2B1, and P-gp.

Transporter (peptide)	Human liver	Freeze-thaw stability	Bench top stability
OATP1B1 (NVTGFFQSFK)	HL1	116.5 ± 0.8	90.5 ± 0.8
	HL2	102.9 ± 3.9	99.3 ± 8.2
	HL3	99.3 ± 11.6	94.3 ± 5.7

OATP1B3 (NVTGFFQSLK)	HL1	98.8 ± 6.9	93.8 ± 10.5
	HL2	109.2 ± 2.9	94.7 ± 5.1
	HL3	93.9 ± 10.7	96.0 ± 4.4

OATP2B1 (VLAVTDSPAR)	HL1	93.9 ± 4.6	91.8 ± 20.0
	HL2	95.7 ± 18.2	97.8 ± 10.9
	HL3	94.4 ± 4.6	91.6 ± 5.1

P-gp (NTTGALTTR)	HL1	95.3 ± 2.2	85.4 ± 6.7
	HL2	102.5 ± 4.6	95.9 ± 6.0
	HL3	99.6 ± 11.9	105.5 ± 12.8

**Table 4:** Comparison of precision of protein quantification using SIL versus SILAC internal standard methods.

Transporter	Mean of standard deviation		Range of standard deviation for the 20 liver samples		*P* value (paired *t*-test)
	SIL	SILAC	SIL	SILAC	
OATP1B1	0.16	0.36	2.3–16.0	3.0–37.6	<0.001*
OATP1B3	0.10	0.25	2.2–27.2	8.2–90.3	0.03*
OATP2B1	0.10	0.10	1.1–14.0	1.2–9.2	0.53
P-gp	0.05	0.07	3.1–21.3	8.0–60.4	0.01*

*The standard deviation of the individual log protein concentrations in each liver sample, estimated using the SIL or the SILAC internal standard method, was compared using the paired *t*-test. *P* < 0.05 was considered significant.

## Abbreviations

LC-MS/MS: Liquid chromatography tandem mass spectrometry
OATP: Organic anion-transporting polypeptide
P-gp: P-glycoprotein
QC: Quality control
SIL: Stable isotope label
SILAC: Stable isotope labeling with amino acids in cell culture
UW: University of Washington
